# Cysteine sulfinic acid and sulfinylated peptides

**DOI:** 10.1039/d5cb00040h

**Published:** 2025-05-09

**Authors:** Laura Hayward, Matthias G. J. Baud

**Affiliations:** a School of Chemistry and Chemical Engineering, University of Southampton Southampton SO17 1BJ UK m.baud@soton.ac.uk

## Abstract

Cysteine sulfinic acid (CSA) is a stable post translational modification in nature. While long considered to be an irreversible by-product of accidental overoxidation of the cysteine sulfur, evidence in the last two decades has accumulated for its role in numerous and tightly regulated mechanisms. Proteomics studies in the last two decades have identified CSA in hundreds of cellular proteins, highlighting its omnipresence at the core of the cysteine redoxome. Elsewhere, structural studies have shed initial light on the molecular mechanisms underlying CSA reduction *in vivo* by the sulfiredoxin (Srx) enzyme. While peroxiredoxins have for a long time been the only known substrates to be turned over by Srx, recent studies have uncovered a plethora of potential new substrates of Srx, opening new avenues of investigation in fundamental biology, but also possibly opening new opportunities for developing novel medicines targeting the redoxome, especially in cancer and neurodegeneration. This review first summarises important knowledge surrounding the stereo-electronics and biochemical properties of CSA, including how it is reduced by Srx. In a second part, it highlights the chemical methods recently developed for CSA characterisation, with important examples of electrophilic probes for CSA covalent adduct formation. Crucially, *in vitro* biochemical studies of CSA and its peptides have historically proven difficult, in great part due to the limitations associated with the few existing synthetic methods available. Here, we also provide a summary of synthetic methods currently available for CSA incorporation into peptides, and their current limitations.

## Abstract 2

Cysteine sulfinic acid (CSA) is a stable post translational modification in nature.

## Graphical abstract

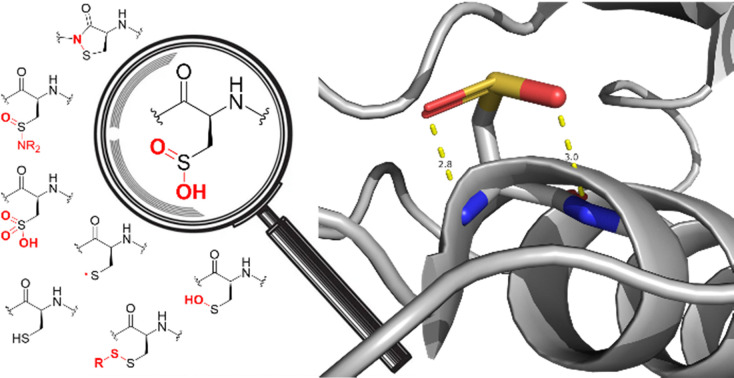

Late stage, post-translational modification (PTM) of amino acids is an ingenious way employed by nature to expand the structural, hence functional diversity of proteins far beyond the 20 canonical amino acids.^1,2^ Such modifications play essential roles in all aspects of biology, with many well-known examples including acetylation, methylation, phosphorylation and glycosylation. The extended range of activities accessible through these modifications have prompted chemists to develop the molecular tools to study their function and dynamics, but also to exploit them for the development of new classes of biologicals and drugs (*e.g.* small molecules, peptidomimetics). In particular, cysteine oxidative post-translational modifications ([O]-PTMs) have attracted increasing interest in recent years, due to cumulative evidence of their important roles in several critical and very complex cellular processes, notably in oxidative stress response mechanisms, aging, and redox cellular signalling pathways.^3^ Cysteine can exist in a variety of chemically and functionally distinct oxidation states ranging from −2 to +6 (Fig. 1(A)), owing to sulfur's ability to expand its octet. *In vivo*, oxidative post-translational modification of cysteine is mediated by various reactive species, such as reactive oxygen species (ROS), nitrogen species (RNS), and sulfur species (RSS). Many of these [O]-PTMs occur *via* either direct nucleophilic attack or an electron transfer mechanism involving the electron rich(er) sulfur of cysteine and a reactive electrophile,^4^ such as hydrogen peroxide (H_2_O_2_) or hydroxyl radicals (HO˙).^5^ The cysteine thiol will react orders of magnitude faster with H_2_O_2_ when in the deprotonated thiolate form (Cys-S^−^),^6^ however, this is not the only factor which governs its reactivity. The microenvironment of the cysteine will also influence reactivity since surrounding amino acids will have differing polarities and interactions. Hence, the p*K*_a_s of protein thiols will vary, ranging from approximately 2.5–12, whereas at physiological pH with no external influences the p*K*_a_ of cysteine lies at around 8–9.^7^ It is estimated that up to 12% of all cysteines in the human proteome are in an oxidised form in cells and tissues.^8^ Numerous states of ROS modified cysteine have been identified to date (Fig. 1(A)), whose cellular levels are tightly regulated by the reversible action of specific enzymes with oxidase or reductase activities. Despite the extensive literature surrounding the omnipresence of ROS modified cysteines *in vivo*,^3,9^ their influence on protein stability and recognition properties is still relatively poorly understood.

**Fig. 1:**
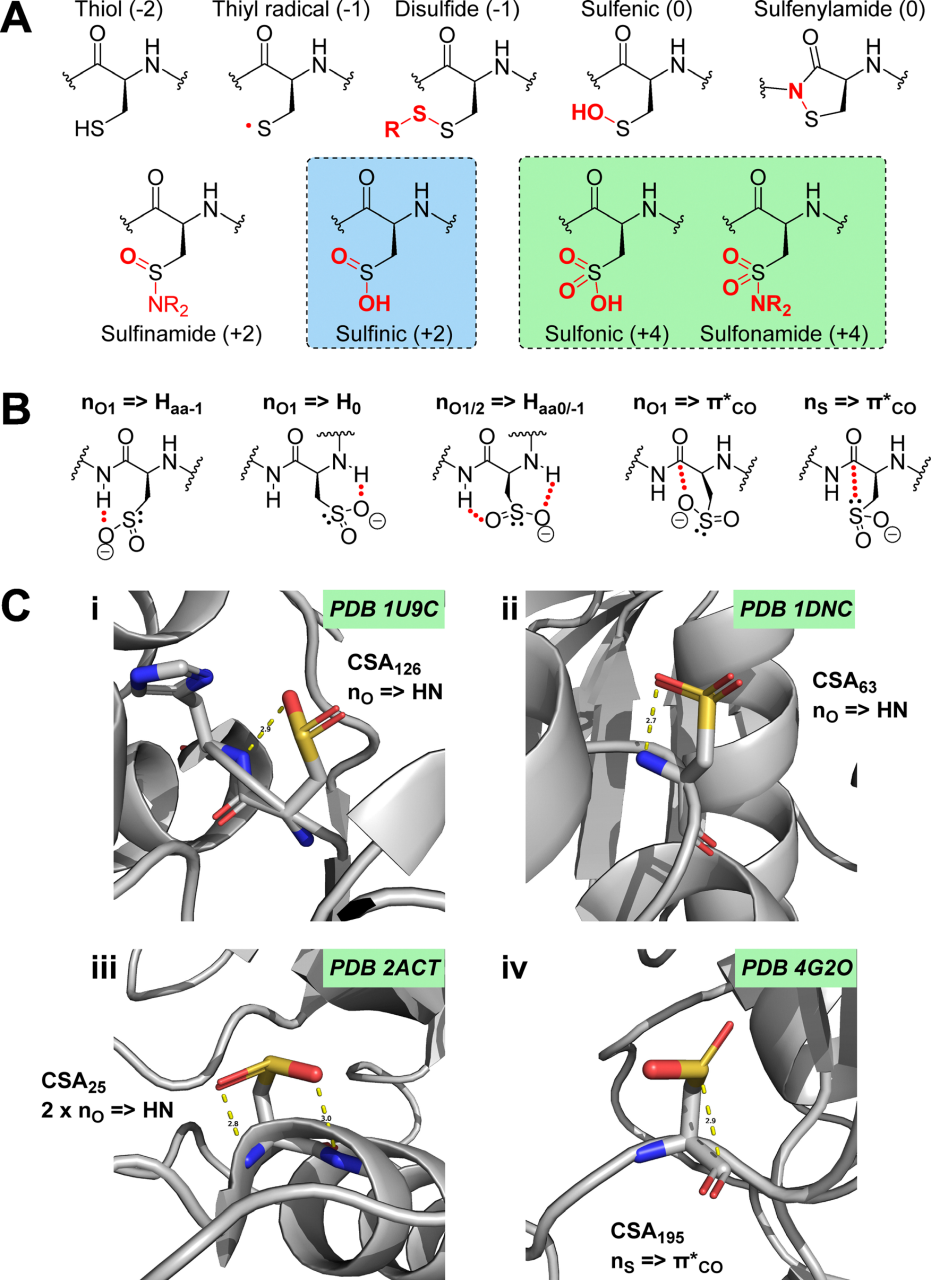
Molecular and recognition properties of CSA. (A) Important ROS induced cysteine modifications of biological relevance (non-exhaustive). The sulfur oxidation state is given in brackets. Modifications that are mostly irreversible are highlighted in green, CSA is highlighted in blue; (B) diverse intramolecular interactions between the sulfinate group and with peptidic backbones, evidenced by computational conformational analyses and Protein Data Bank (PDB) mining; (C) representative examples of such interactions observed in the PDB. H-bonding and n_S_ → π*_CO_ interactions are shown as yellow dashes, with distances between both heteroatoms highlighted.

## Biochemistry of cysteine sulfinic acid (Cys-SO_2_H, “CSA”, oxidation state +2)

CSA is arguably among the most elusive cysteine Oxi-PTM, and possesses several distinctive electronic and steric characteristics.^10–13^ CSA is negatively charged at physiological pH (p*K*_a_ ∼ 2). Despite its negative charge, it has unique properties compared to aspartic or glutamic acids. It has Lewis basic character at both the sulfur and oxygens, *via* their lone pairs, making it weakly nucleophilic and often acting as a soft nucleophile. In contrast to the carboxylic acid moiety, which is trigonal planar (bond angles ∼120°), the sulfinic acid is tetrahedral around sulfur (bond angles ∼107°) owing to the lone pair on the sulfur. Mass spectrometry of molecules containing cysteine sulfinic acid usually show the [M + H]^+^ molecular ion, though it is also common to observe the dehydrated [M + H − H_2_O]^+^, resulting from the loss of a water molecule.^14,15^ The presence of the sulfur, combined with the tetrahedral geometry also makes the sulfinic acid significantly larger sterically than its carboxylic counterpart. Computational studies by Urmey and Zondlo on a subset of approximately 400 CSA containing structures identified in the Protein Data Bank (PDB) also highlighted conformational preferences of CSA, where both the oxygen and sulfur of the sulfinate group engage in intra-molecular hydrogen bonding with their own backbone N–H, and that of the *n* ± 1 amino acid. The same studies also supported n → π* interactions between the CSA lone pairs at both O and S with the antibonding orbital of their backbone amide, in line with the Lewis basicity of sulfinates (Fig. 1(B)).^16^

Astonishingly, CSA alone has been estimated to account for ∼5% of accessible (*i.e.* not buried in a protein core) cysteines in the human proteome.^17^ Sulfinic acids are far less reactive than sulfenic acids or disulfides, hence CSA is a chemically stable modification in peptides/proteins. Increasing evidence in recent years have suggested that CSA acts as a regulatory modification,^3,9,18^ though the full scope of its biological activity and regulation remains poorly understood. While CSA was long thought to be an irreversible mark in humans,^17,19^ recent findings have pointed to a role for sulfiredoxin (Srx) in CSA reduction.^20,21^ Oxidation to CSA has been shown to modulate protein signaling in diverse ways, leading to either gain- or loss-of-function (GOF/LOF), depending on the protein itself, and the site of oxidation. First examples of modulation of protein function by CSA were reported almost three decades ago. In the dark, the active site iron of the bacterial enzyme nitrile hydratase (NHase)^22^ is in an inactive state, and is tightly bound with an endogenous nitric oxide (NO) molecule. Photo-dissociation of NO leads to NHase activation and catalytic hydrative activity toward its nitrile substrates.^23–25^ Mass spectrometry and structural studies of NHase from *Rhodococcus erythropolis* highlighted the importance of the oxidation state of C112 and C114 in the active site, where their spontaneous aerobic oxidation to sulfinic and sulfenic acids respectively, allows H-bonding with proximal and conserved R141 and R56.^26,27^ Combined with Fe complexation *via* their respective sulfur atom, they are thought to critically stabilise the structure of the active site so called “claw setting”, allowing catalysis. In contrast, Murakami and co-workers shows that reconstitution under anaerobic conditions (argon atmosphere) yields an inactive NHase, with gradual recovery of catalytic activity upon spontaneous aerobic oxidation of C112 to the corresponding sulfinic acid.^28^ The human protein DJ-1, also known as Parkinson disease protein 7 and encoded by the PARK7 gene in human, inhibits cellular apoptosis in its oxidized form (C106 → CSA106) and has protective functions in Parkinson's disease.^29–32^ Elsewhere, oxidation to CSA in matrix metalloprotease-7 (MMP-7) activates its protease activity, partly *via* the loss of key sulfur–zinc interactions as a result of sulfur oxidation.^33^ On the other hand, the phosphorylation-dependent prolyl isomerase Pin1 loses its isomerase activity upon oxidation of its active-site cysteine to CSA.^34^ Elsewhere, CSA has been shown to modulate the electronic/binding properties and catalytic activity of several cobalt containing metalloenzymes, by coordinating their active site cobalt.^35^ The bacterial thiocyanate hydrolase (SCNase) hydrolyses thiocyanate to carbonyl sulfide and ammonia, and share important sequence similarities with NHase. CSA also plays a central role in protein homeostasis in diverse organisms. For example, in plants, normoxic oxidation of N-terminal Cys of target proteins by protein cysteine oxidases (PCOs) makes them substrates to arginylation and degradation *via* Cys N-degron pathways.^36,37^ Conversely, hypoxia reduces PCO activity, leading to protein stabilisation.

## Reduction of sulfinylated peroxiredoxins by sulfiredoxin

**Fig. 2:**
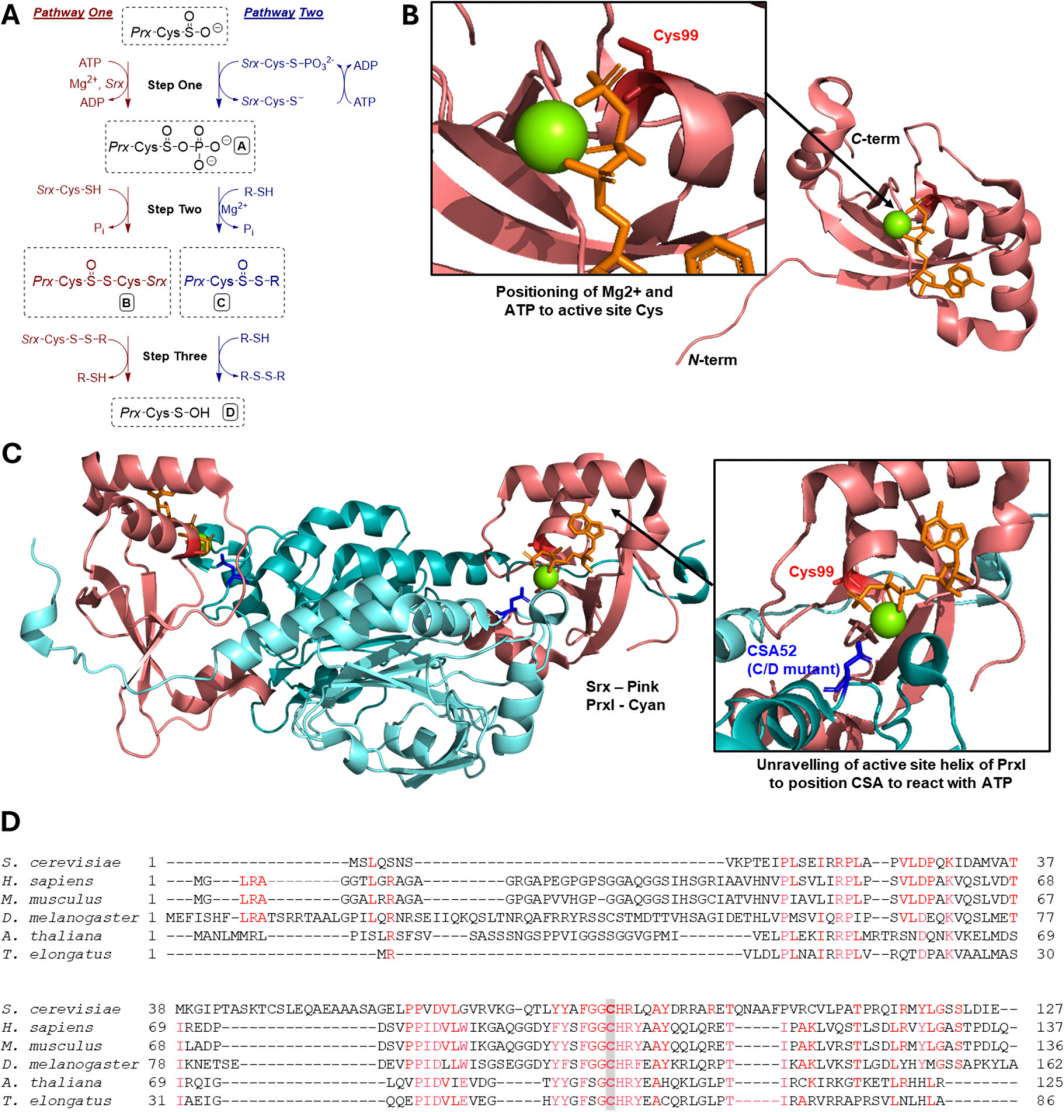
(A) Previously proposed models for reduction of CSA by Srx: pathway one initially presented by Biteau *et al.*,^38^ Pathway two by Jeong *et al.*^20^ (B) Crystal structure of human Srx (hSrx) in complex with ATP and Mg^2+^(PDB: 3CYI).^47^ (C) Crystal structure of hSrx in complex with PrxI, ATP and Mg^2+^ (PDB: 3HY2, with mutation of Prx CSA to Asp to avoid reaction with Srx).^48^ (D) Sequence conservation of sulfiredoxin across various species. Conserved residues are shown in red. The catalytically conserved active site C99 (homo sapiens numbering) is highlighted in grey.

First identified in yeast in 2003, the sulfiredoxin enzyme (Srx, 14 kDa) belongs to the wider family of oxidoreductases, and is conserved across higher eukaryotes.^38–41^ Srx has been known since to reduce CSA in only a handful of substrates *in vivo*, notably peroxiredoxin (Prx) enzymes following overoxidation/inactivation during oxidative stress.^42,43^ Prx antioxidant activity is important for cellular redox homeostasis and regulating the level of diverse reactive oxygen species (ROS), especially H_2_O_2_.^44,45^ The precise molecular mechanism underpinning CSA reduction by Srx has been debated but is thought to proceed *via* ATP/Mg^2+^ dependent sulfinate phosphorylation/activation (step 1) and formation of a covalent Prx–Srx complex (step 2). Reaction of the thiosulfinate with a secondary thiol (*e.g.* glutathione or thioredoxin) then liberates the Srx disulfide and Prx sulfenic acid products (step 3), whose fate is then determined downstream depending on the cells redox balance and microenvironment (Fig. 2(A)).^41^ Strikingly until 2018, Prx1-4 were the only known/validated substrates of human Srx.^39,40,46^

**Fig. 3:**
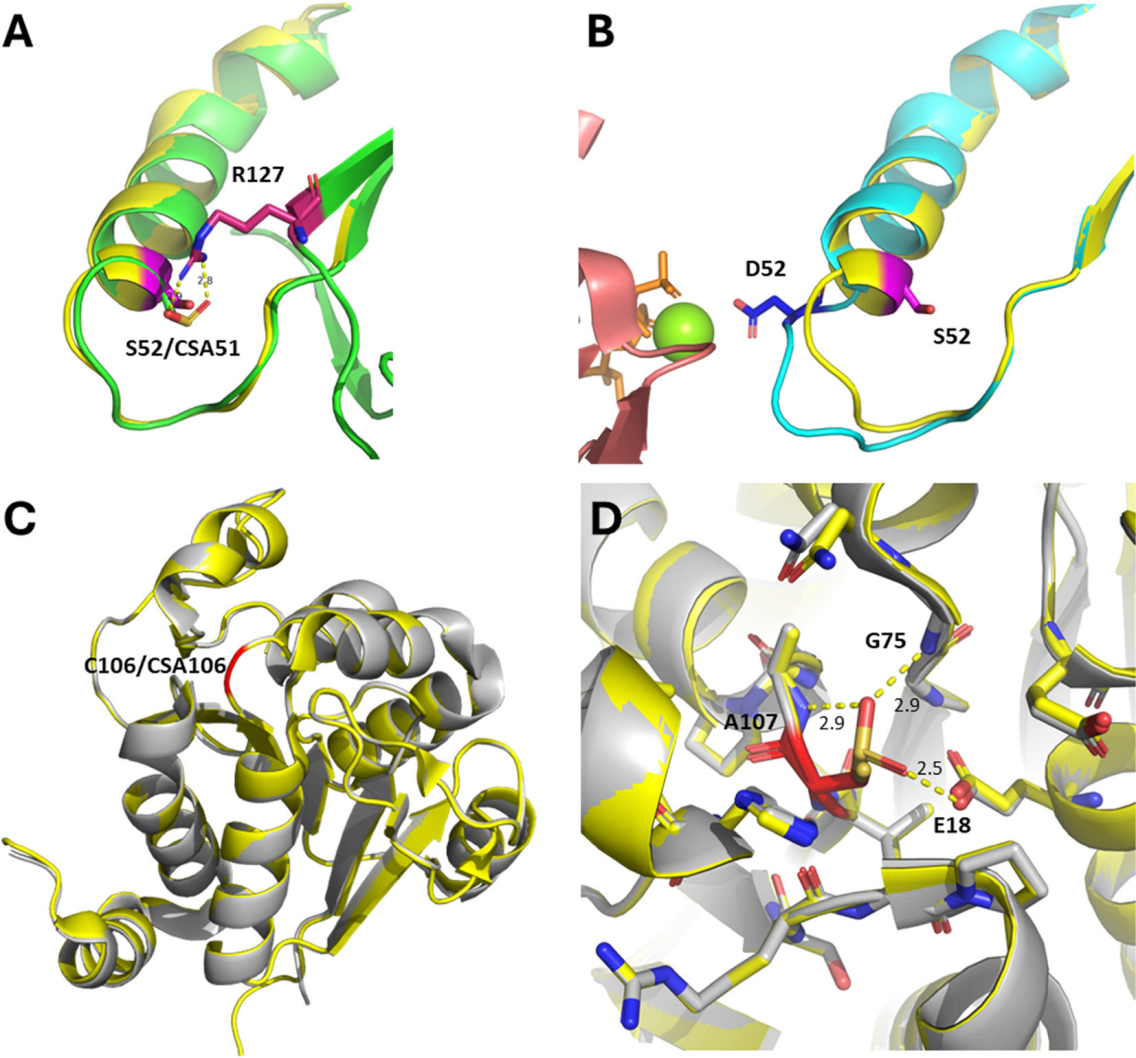
(A) Superimposition of the crystal structures of unbound Prx1 (yellow cartoon, C52 to S52 mutant, PDB 7WEU^50^) and unbound sulfinylated Prx2 (green cartoon, CSA51, PDB 1QMV^51^). Position 52 (Prx1 numbering) is highlighted in magenta. R127 (Prx2 numbering) is shown in dark pink, and hydrogen bonds with CSA51. (B) Superimposition of the crystal structures of unbound Prx1 (yellow cartoon, C52 to S52 mutant, PDB 7WEU) and Prx1 (cyan cartoon, C52 to D52 mutant, PDB 3HY2^48^) bound to Srx (salmon cartoon, Mg^2+^ is shown as a green sphere, ATP is shown as orange sticks). Position 52 is shown in magenta and dark blue respectively, highlighting local unfolding and movement of the beta-carbon by approximately 8 Å. (C) Superimposition of the crystal structures of WT DJ-1 (yellow cartoon, PDB 4ZGG) and sulfinylated DJ-1 (grey cartoon, PDB 1SOA,^29^ CSA106) highlights conservation of the protein fold. Position 106 is shown in red. (D) Same alignment, with close-up on position 106. Amino acids within 5Å of C106/CSA106 are shown as sticks. A107, G75, and E18 engage in H-bonding interactions with CSA106, and are labelled.

Recent mechanistic and structural analysis of Srx:Prx complexes have revealed that in apo non-oxidised (*i.e.* thiol state) Prx1, C52 is flanked by hydrophobic F50 and V51, and is mostly buried. Structural studies of Prx2 have shown that the sulfinate (CSA51 in Prx2) engages in polar interactions with the neighbouring R127 (Fig. 3(A)). R127 is conserved in Prx1 (R128). Complex formation with Srx leads to the CSA52 containing helix to locally unfold and move by ∼8 Å, positioning the sulfinate within ∼5 Å of the γ-phosphate of the ATP molecule bound in the Srx active site (Fig. 2(B) and (B)).^48^ Srx:Prx covalent complex formation (C99_Srx_–C52_Prx1_, PDB 7LJ1^49^) leads to the burial of >500 Å^2^ at each Srx–Prx active site interface. In this complex, a “concave” hydrophobic surface formed by the side chains of L53/L82/F96/V118/Y128 of Srx engages in extensive hydrophobic contacts with a “convex” hydrophobic patch centred on a F50/V51 motif adjacent to C52 of Prx1 (Fig. 2(B) and (C)). This is consistent with recent site directed mutagenesis (SDM) experiments showing that binding (*K*_d_ = 7.0 μM) and reduction of Prx1 is drastically reduced by hydrophobic to charged mutations of the Srx hydrophobic surface.^49^ The precise molecular mechanism underpinning such local rearrangement, however, remains poorly understood.

In contrast, unoxidised DJ-1 (C106 thiol, PDB 4ZGG) displays essentially the same fold and conformation upon oxidation of the partly solvent exposed C106 to its corresponding CSA (PDB 1SOA^29^) (Fig. 3(C)). In this structure, one oxygen atom of the sulfinate group engages in hydrogen bonding with the protonated side chain of E18,^52^ while the other oxygen does hydrogen bond with the backbones of G75 and A107 (Fig. 3(D)). These additional interactions contribute to the higher thermal stability of the sulfinylated DJ-1 (*T*_m_ ≈ 77 °C) compared to wild-type DJ-1 (*T*_m_ ≈ 64 °C).^53^ Witt and co-workers have previously discussed the impact of the Glu18 protonation state on the depressed p*K*_a_ of C106, in part explaining its propensity to oxidation.^52^

Historically, several closely related models have been proposed for the mechanism by which this reduction occurs (Fig. 2(A)).^20,38^ Jonsson *et al.*^47,48^ confirmed the mechanistic steps initially presented by Biteau *et al.* (pathway one), using various crystal structures. The crystal structure of human Srx (hSrx) in complex with ATP and Mg^2+^ (PDB: 3CYI, Fig. 2(B)), showed that the reduction must proceed directly *via* a sulfinic phosphoryl ester intermediate (Structure A, Fig. 2(A)). The Mg^2+^ positions the active site ATP such that the γ-phosphate of ATP is orientated towards solvent molecules, preventing possible in-line attack from the Srx active site Cys (hSrx-Cys^99^), with loss of such orientation shown upon removal/replacement of the magnesium ion.^47^ The crystal structure of hSrx in complex with PrxI, ATP and Mg^2+^ (PDB 3HY2, with mutation of Prx CSA to Asp to avoid reaction with Srx, Fig. 2(C)) allows visualisation of the structural changes that lead to the attack on ATP.^48,54^ These changes include: the unfolding of the C-terminus on to the backside face of Srx; the unfolding of the active site helix of Prx; and the displacement of the conserved YF motif from the Prx active site which blocks approach of the ATP bound Srx.^54^ These changes are thought to be mediated by the interaction of the hydrophobic surface of Srx with a conserved Phe^53^ residue of PrxI which orientates the CSA of Prx to allow for in-line attack of the γ-phosphate.^54^ Jonsson *et al.* suggest that the reduction of CSA requires further stabilisation using the C-terminus of Prx (residues 172–186 of PrxI – conserved) interacting with the backside surface of Srx, in addition to typical active site interaction. The formation of the covalent Prx–Srx complex, linked by a thiosulfinate bond (Structure B, Fig. 2(A)) has been characterised by Roussel *et al.*^55^ and that its subsequent reduction requires an additional reducing agent such as DTT (1,4-dithiothreitol) or Trx (step three). Therefore, pathway one is widely accepted as the correct mechanistic pathway.

Previously, it has been suggested that Srx only reduces CSA moieties present in 2-Cys Prxs. However, chemical proteomics studies by Akter *et al.* in 2018 revealed ∼55 potential new targets for Srx, not related to the Prxs.^46^ These recent insights into new targets of Srx has opened a new realm of potential regulatory functions of CSA and Srx, that are yet unknown. Currently, there is no published literature considering what specifically links these substrates functionally and/or functionally, what makes them prone to oxidation to CSA, and how they might commonly interact with Srx. However, we anticipate that the discovery of such a link will give a greater understanding of this area and eventually allow *de novo* identification of new targets and possible functionalities of Srx.

To date, hundreds of sulfinylated proteins have been identified in humans.^46,56,57^ However, for many of these proteins, it remains to be shown whether these sulfinylation marks are part of specific and regulated signaling networks, or merely by-products of unspecific oxidative stress. Additionally, while a small subset of these proteins are validated substrates of Srx, notably Prxs and to some extent DJ-1, the majority remain to be unambiguously confirmed. Thorough *in vitro* characterisation of their protein–protein interactions using biochemical/biophysical and structural methods will be paramount for the functional elucidation of their complexes. This will in great part depend on two main aspects, namely the availability of robust chemical tools to reliably detect CSA in the biological environment, and generalisable synthetic methods to prepare sulfinylated peptides for biochemical studies. The next sections discuss the chemical biology of CSA, summarizing current chemical probes for CSA detection, in addition to the synthetic methods available for the preparation of sulfinylated peptides.

## Chemical biology, CSA targeted electrophilic probes for detection

**Scheme 1:**
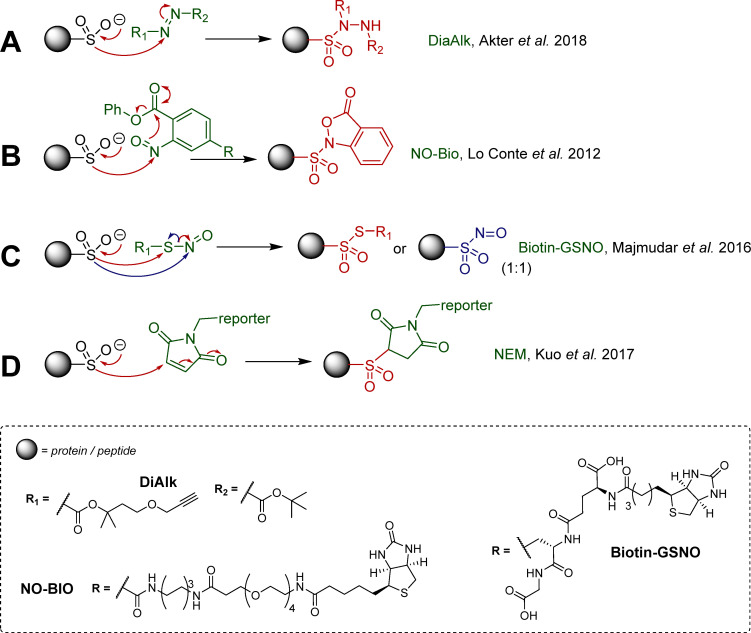
Important examples of electrophilic reagents developed for use in analysis of *S*-sulfinylation, (A) diazene probes (DiaAlk);^46^ (B) *C*-nitroso compounds (NO-Bio);^12,59^ (C) *S*-nitroso compounds;^60^ (D) maleimide based Michael acceptors.^61^

Methods to detect cysteine sulfinic (+2) and sulfonic (+4) acids, despite their extended lifetime, are scarce due to their decreased nucleophilicity, relative to the lower oxidation state PTMs (*e.g.* cysteine thiol), making the design of selective electrophilic probes challenging. Antibodies against sulfonylated and sulfinylated Prx have been reported,^58^ though their affinity and specificity have been debated. A number of electrophilic nitrogen containing small molecules have been reported by the Carroll lab, which lead to selectively identifiable adducts with sulfinic acids. The bifunctional DiaAlk probe (Scheme 1(A)) is an alkyne derivative of di-*tert*-butyl azodicarboxylate (DBAD), which covalently modifies *S*-sulfinylated proteins and allows detection of the resulting sulfonylated hydrazine adduct.^46^ A variant of DiaAlk known as DiaFluo was also reported for fluorescent imaging of proteins. The sulfinic acid reacts with the electrophilic probe to form a sulfonohydrazide linkage, which is markedly more stable than adducts from thiols, which can readily undergo exchange. Detection of sulfinic acids was reported by Carroll and co-workers using aryl nitroso derivatives (Scheme 1(B)). In this process, nucleophilic attack of the nitroso by the sulfinate generates an *N*-sulfonyl hydroxylamine (which is labile), which further reacts intramolecularly with the ester group at the *ortho* position to generate the corresponding and stable *N*-sulfonylbenzisoxazolone, with concomitant elimination of methanol (Scheme 1(B)). Conjugation with biotin lead to bifunctional probe NO-Bio for affinity capture, which was used for cellular quantification of CSA in diverse cell lines in comparative sulfinylome profiling studies. Of note, this procedure still required prior cysteine thiol capping to prevent cross-reactivity.^12,59^ Nevertheless, this first-in-class probe showed preferential reactivity for CSA using both purified proteins and live cells, demonstrating the potential for selective targeting of CSA despite its lower nucleophilicity. More recently, Martin and co-workers reported affinity probe biotin-GSNO, whose *S*-nitrosothiol forms thiosulfonate linkages upon reaction with sulfinates (Scheme 1(C)).^60^ However, this methodology, similarly to that reported by Carroll, still requires prior capping of free thiols (with iodoacetamide), to prevent nucleophilic attack and cleavage of the thiosulfinate. The latter was also found to be sensitive to reduction by tris(2-carboxyethyl)phosphine (TCEP), emphasising the importance of experimental conditions but also offering a simple way for release. Prototypical biotin-GSNO has been employed for CSA enrichment, and to characterise the sulfinylation of DJ-1 at position 106. Despite its usefulness, the lability of the thiosulfinate linkage has limited the application of this methodology. Partly addressing this issue, the Martin group recently reported the reaction of *N*-ethylmaleimide (NEM) with aryl and alkyl sulfinic acids in aqueous buffer, leading to the formation of the sulfonyl-succinimide (Scheme 1(D)).^61^ Again, the procedure requires blocking of the reactive free thiols prior to NEM treatment. Importantly, the sulfonyl-succinimide conjugate is stable for analysis below pH 6, but degrades at higher pH. This approach was employed for CSA characterisation in purified DJ-1 protein and mammalian HEK-293T cell lysates.^61^ Of these probes, DiaAlk seems overall to offer the most promise, having delivered a wide range of new data on putative new targets of Srx, along with the significant advantage not to require extensive capping of free thiols.

## Synthetic methods toward sulfinylated peptides

**Scheme 2:**
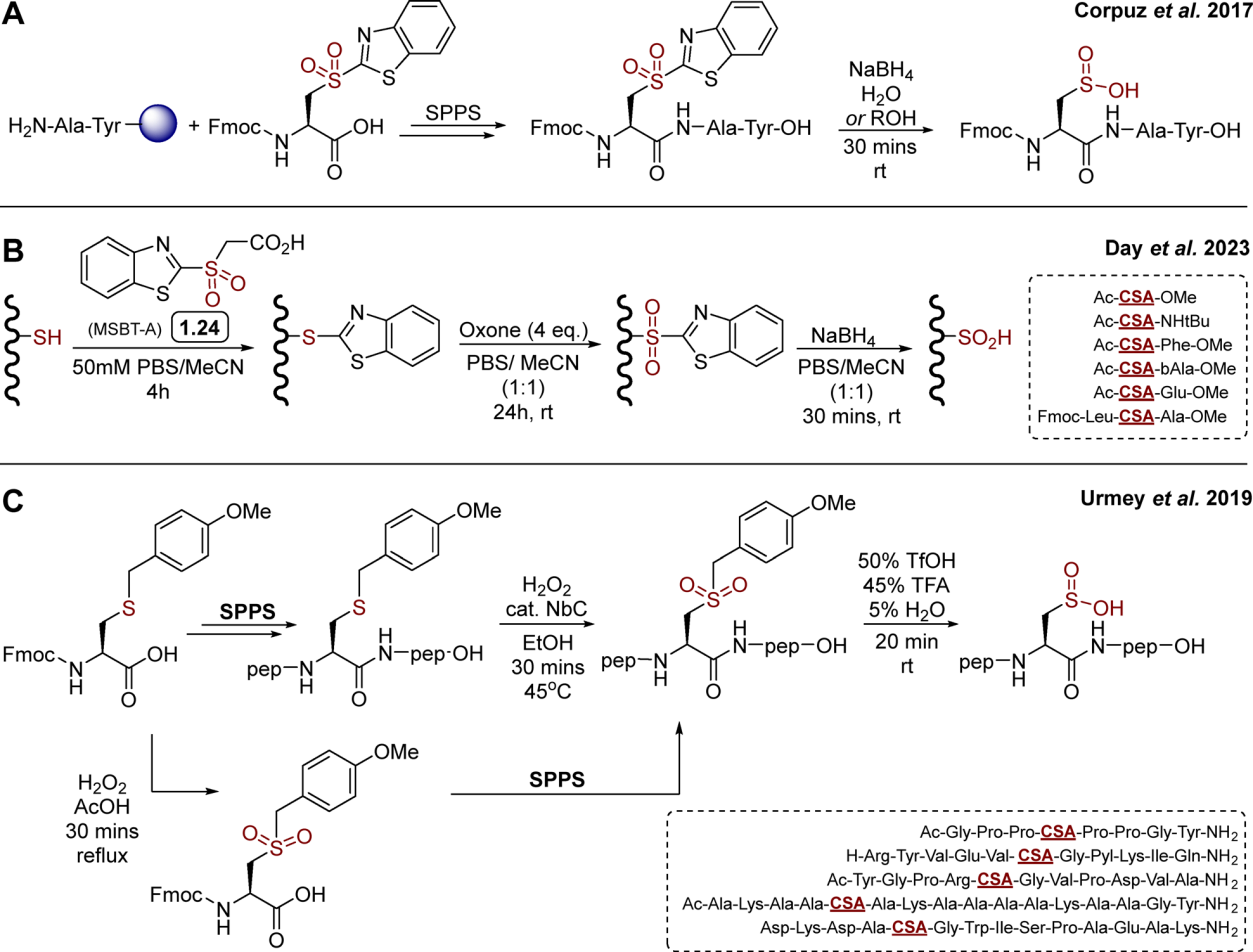
Reported SPPS based synthetic routes towards CSA containing peptides. (A) Corpuz's strategy toward a model CSA-Ala-Tyr tripeptide, employing a 2-sulfonylbenzothiazole functionalised cysteine as SPPS compatible precursor of CSA;^71^ (B) day's variation based on late stage modification of short cysteine containing peptides by S_N_Ar protection – oxidation – nucleophilic deprotection; (C) Urmey's strategy using an acid cleavable 4-methoxybenzyl protecting group.^75^

Relatively few synthetic methods have been reported in the literature for the synthesis of sulfinylated peptides. Historically, first strategies were based on direct oxidation of cysteine thiol in H_2_O_2_ containing solutions, leading to mixtures of oxidation states of the target peptides, which can eventually be separated by chromatography, though in generally low yields. Elsewhere, Urmey and colleagues prepared four short peptides with CSA embedded within an Ala rich sequence, for conformational studies.^16^ They employed a similar approach for the modification of a key metal-binding aspartate in a canonical 14-mer EF-Hand motif (**D**K**D**A**D**GWISPAEAK) to Cys/CSA. The resulting Cys *vs.* CSA containing probes exhibited differential binding of terbium(iii) and induction of luminescence, providing a new, prototypical tool to study CSA formation.^62^ A number of thiol dioxygenase (TDO) enzymes^63^ that catalyse the oxidation of thiols to sulfinic acids using molecular oxygen have been characterised, including mammalian cysteine dioxygenase (CDO) and 2-aminoethanetiol (cysteamine) dioxygenase (ADO), which are involved in sulfur metabolism.^64–69^ While important aspects of their biochemistries have been uncovered several decades ago, examples of their use for the synthesis of sulfinylated peptides or full proteins is limited. N-terminal Cys to CSA oxidation by TDOs has been observed in plant, catalysed by plant cysteine oxidases (PCOs).^36,37^ N-Terminal Cys oxidation plays a key role in selective proteolysis *via* an oxygen-dependent branch of the N-end rule pathway. A similar ADO-mediated process was also identified in mammalian cells.^70^ The current structural and biochemical knowledge of TDOs has been summarised very recently by Perri and Licausi in an excellent review.^63^ While the N-terminal regioselectivity of CDOs may present advantages for further development of enzymatic methods to produce N-terminally sulfinylated peptides, current gaps in knowledge of their catalytic substrate recognition properties have hampered their generalisation. Recently, the focus has shifted toward the development of more selective reagents and procedures. Corpuz and Schwans reported a 2-sulfonyl benzothiazole (2-SBT) derivative as an SPPS compatible building block (Scheme 2(A)).^71^ This 2-SBT protected sulfone is a CSA precursor, and can be readily synthesised in three steps from l-cysteine (51%, *ca.* 200 mg). The benzothiazole acts as a protecting group and can be cleaved by S_N_Ar using excess sodium borohydride in water/alcohol mixtures to reveal the target sulfinate. Using Wang resin and standard HBTU/DIPEA coupling in DMF, the authors reported the synthesis of the model peptide H_2_N-**CSA**-Tyr-Ala-OH, after TFA mediated cleavage and subsequent nucleophilic deprotection of the BT protecting group with NaBH_4_. Yield, scale and enantiopurity of the product were not mentioned, and the study was limited to this one example. It is also worth noting that the CSA precursor was introduced at the N-terminal position (*i.e.* last), hence it is unclear whether the BT protecting group would survive intermediate steps, especially Fmoc deprotection with 20% piperidine/DMF. Heteroaryl sulfones, including 2-sulfonyl benzothiazole derivatives, are known to be labile in the presence of diverse nucleophiles.^72–74^ Nevertheless, this proof-of-concept study highlighted the 2-SBT motif as a useful precursor for synthetic access to short, N-terminally sulfinylated peptides.

In 2023, Day and co-workers reported a related approach based on MSBT-A (Scheme 2(B)) for the late-stage oxidation of cysteine (free thiol) in model di- and tri-peptides.^76^ MSBT-A is functionalised with a carboxylic acid on its exocyclic side chain, making it similarly reactive *via* S_N_Ar though more soluble in aqueous buffer than previously reported non-functionalised SBT derivatives, requiring over 20% acetonitrile as co-solvent to reach micromolar concentrations in aqueous buffer.^72^ This three-step, operationally simple sequence relies on (i) cysteine thiol arylation by S_N_Ar with MSBT-A, (ii) oxidation with oxone, and (iii) final nucleophilic deprotection with NaBH_4_ (Scheme 2(B)). Despite reaction times of up to 24 hours, oxone proved effective for the oxidation of the thioether intermediate, providing the corresponding sulfone in high yields. This mild oxidation process proved to be compatible with diverse amino acid side chains, apart from methionine which was competitively oxidised to the corresponding sulfone. While the side chains of the target model peptides were carefully chosen to be oxidation insensitive (*e.g.* Phe, Leu, Ala, β-Ala, Glu), the late-stage applicability and feasibility to embed CSA within a short sequence (*versus* N-terminal sulfinylation only, *vide supra*) represents a notable expansion of the scope of this chemistry toward library generation. This three steps sequence was also applied to model protein bovine serum albumin (BSA), which contains 17 disulfide bonds and a single, surface exposed cysteine (Cys58). Following trypsin digestion, LC–MS/MS based proteomics analysis showed partial formation of CSA58, although BSA folding/stability and potential off-target modifications were not mentioned. While preliminary, this represents a first step toward more general methodologies for the late stage sulfinylation of protein substrates.

In 2019, Urmey *et al.*^75^ employed a 4-methoxybenzyl (PMB) functionalised sulfone as CSA precursor to synthesise three longer model peptides (8-, 11- and 12-mer, Scheme 2(C)). Following incorporation of the commercially available Fmoc-Cys(Mob)-OH by SPPS and cleavage from the resin, the *S*-PMB cysteine thioether intermediate was oxidised using H_2_O_2_ and catalytic niobium carbide (NbC) to the corresponding sulfone (Scheme 2(C)). Finally, the Mob group was cleaved in acidic conditions to reveal the sulfinic acid. The PMB group proved surprisingly stable, and required forcing conditions for deprotection, which was eventually successful using a 50 : 45 : 5 TfOH/TFA/H_2_O mixture. This methodology allowed access to Pro rich sequences, and was compatible with more polar residues such as Tyr, Arg/Lys, Asp/Glu/Gln, or non-canonical pyrrolysine. However, and similarly to the MSBT-A based method (*vide supra*), the late-stage oxidation of the full peptides does not allow for oxidation sensitive amino acids such as Cys, Met and Trp to be included in the sequence. As a possible way to overcome this challenge, the authors prepared and incorporated a pre-oxidised Fmoc-Cys(mob)-OH. Despite the need of forcing coupling conditions on a single example, this alternative route may offer a path to an expanded range of potential sequences, including oxidation sensitive motifs. While the overall yields, scalability and stereochemical considerations around potential acid/temperature mediated racemisation of these synthetic methods have not been reported, these will be critical aspects to consider in the future generalisation of these synthetic methodologies. While a general method for the preparation of sulfinylated peptides on scale is still lacking, these seminal studies certainly set a base for further developments.

## Perspective

Despite having been known for decades, CSA has long been considered as a mere by-product of unselective oxidative stress, and only relatively recently has it received more attention. Biochemical and structural studies have shed light on its properties, including its stero-electronics and reactivity, and influence on protein folding. Characterisation of the sulfiredoxin enzyme in the early 2000s has been pivotal in shifting the status of CSA from an oxidation by product, to a regulatory redox post-translational modification. This has been underscored by its implication in a variety of oxidative stress related disease states, notably various cancer types.^21,77–81^ Overall, CSA still remains an underexplored cysteine PTM *in vivo*, and the specificities by which it is generated, but also reduced by Srx in cells is still relatively poorly understood. Beyond sulfinylated Prx and DJ-1, there is little certainty on other substrates. While biochemical and structural studies of Srx:Prx complexes to date have highlighted some important local conformational rearrangements of sulfinylated Prxs to make CSA accessible to the active site of Srx, it is unlikely to be the full picture and currently it is unknown whether this is Prx specific or whether it reflects an overarching mechanism for recognition and catalysis. Structural studies of Srx in complex with sulfinylated Prx have highlighted important interactions mediating complex formation, notably between the hydrophobic surface formed by L53/L82/F96/V118/Y128 of Srx and the F50/V51 hydrophobic motif of Prx1. These favourable hydrophobic contacts are thought to play an important role in the unfolding of the Prx1 helix containing C52 and subsequent approach of the sulfinate to the ATP molecule. The precise thermodynamics underlying complex formation, however, remain only partly understood.

**Fig. 4:**
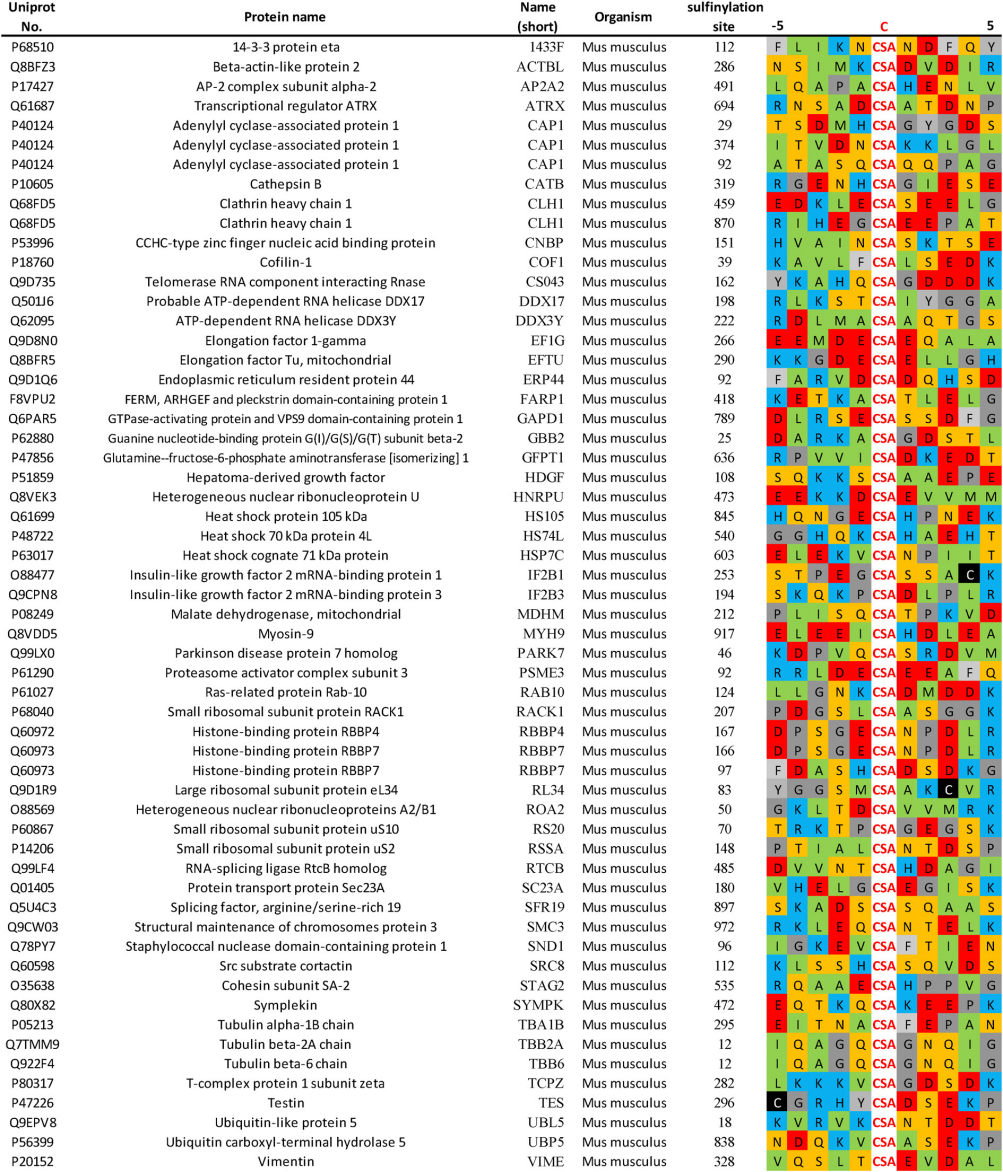
Potential Srx substrates identified by Akter and colleagues in mouse embryonic fibroblasts.^46^ The identified site of sulfinylation (residue 0, red) and surrounding amino acids i − 5 to i + 5 are shown. Residues are colour coded based on side-chain properties: hydrophobic (green), aromatic (light grey), anionic (red), cationic (blue), amide/alcohol (orange), thiol (black), other (dark grey).

**Fig. 5:**
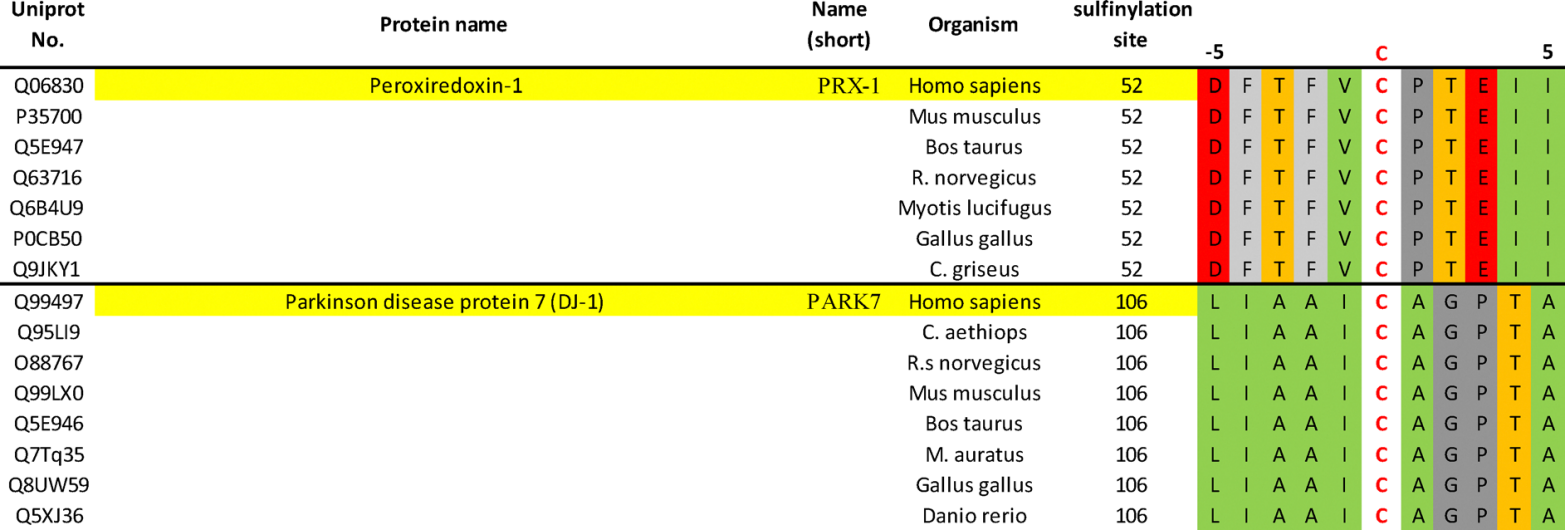
Sequence conservation of PRX-1 and PARK-7 across organisms around their known sulfinylation sites. Alignment of peroxiredoxin-1 and Parkinson disease protein 7 sequences shows conservation across diverse organisms. The site of sulfinylation (residue 0, red) and surrounding amino acids i − 5 to i + 5 are shown. Residues are colour coded based on side-chain properties: hydrophobic (green), aromatic (light grey), anionic (red), alcohol (orange), other (dark grey).

Recently, Akter used the Dialk probe to study differences in sulfinylation in Srx competent (Srx +/+) and Srx KO (Srx −/−) mouse embryonic fibroblasts (MEFs), following induced oxidative stress by H_2_O_2_.^46^ Comparison of sulfinylated protein pools before and after recovery identified over 50 differentially sulfinylated substrates potentially reduced by Srx. Sequence alignment (centred on CSA) of these putative Srx substrates does not highlight any obvious sequence similarity in the direct vicinity of the sulfinylation site (Fig. 4). While more research will be needed to unambiguously validate these proteins as substrates of Srx, these initial results may suggest that Srx does not recognize a specific epitope, but rather involves more complex recognition events spanning distinct secondary/tertiary structural features. It is also worth noting that while this work was carried out in MEFs, mouse Srx and human Srx share a highly similar sequence (Fig. 2(D)). The sequence of Prx1 and DJ-1 around the sulfinylation site is conserved across diverse organisms (Fig. 5), to some extent suggesting that a conserved regulatory mechanism of action. This suggests that they will likely exhibit similar substrate recognition profiles, and that substrates identified in MEFs should also be scrutinized in human cells. Crucially, unambiguous validation of these potential Srx substrates will most likely require their systematic recombinant expression for *in vitro* biochemical and structural studies. It will be key to determine the binding affinity and underlying thermodynamic parameters underlying their binding to Srx, in addition to the associated reduction kinetics in enzymatic assays. While progress has been made in preparation of short sulfinylated peptides, notably using masked CSA precursors in SPPS, much remains to be done to expand the so far narrow synthetic scope of these methodologies.

Accessing site-specifically sulfinylated full-length proteins, while critical to advancing our understanding of the “sulfinylome”, is undoubtedly the biggest challenge currently. Asp has been previously used as a CSA surrogate for structural studies of Srx–Prx complexes.^49^ However, the significant steric and electronic differences between the carboxylate and sulfinate groups make Asp a non-ideal replacement. Genetic code expansion approaches based on *e.g.* AMBER suppression may represent a way forward to access site-specifically sulfinylated full-length proteins. However, neither CSA or a masked derivative have been genetically encoded to date. Despite not being applicable to full-length proteins, synthetic methods to prepare sulfinylated peptides are steadily expending the scope of sequences accessible, and will prove increasingly valuable once generalisable and scalable (tens to hundreds of milligrams). First, sulfinylated peptide libraries will be useful to study the general physico-chemical properties of CSA in a sequence dependent manner *in vitro*, for example to quantify the influence of CSA on local hydrophilicity, molecular interactions (*e.g.* H-bonding, coulombic), and ultimately derive trends for global protein folding and stability. Second, this will aid our understanding of how Srx recognizes its sulfinylated substrates on a molecular standpoint. While Srx substrates may not be recognized sequence specifically (*vide supra*), synthetic access to libraries of sulfinylated peptides will still be valuable to study recognition, *via* systematic assessment of their binding affinity to, and catalytic turnover by, Srx. Such a “fingerprint” will be important not only to rationalize known/existing substrates, but also perhaps for *de novo* prediction of unknown substrates (*e.g. via* BLAST searches).

One important step forward in developing SPPS compatible preparative techniques will likely be the identification of new protecting groups of the sulfinate. These groups will need fine-tuned properties, including sufficient stability to survive coupling and deprotection steps during peptide chain elongation, along with being labile enough to be cleaved in standard “global” deprotection steps. Diverse families of reactivity adjustable heteroaryl sulfone derivatives have been reported, such as 2-sulfonyl-benzothiazoles,^72^ pyridines,^73^ and pyrimidines,^74,82^ among others. Their intrinsic S_*N*_Ar reactivity can be adjusted over many orders of magnitude, allowing chemo- and sometime regio- specific cysteine arylation of cysteine side chains in peptides and protein, concomitant with release of the sulfinate leaving group. These seem well-positioned for future developments. To date, the high electrophilic reactivity of 2-methylsulfonyl BTs has been a challenge, likely in part explaining why most methodologies employing BT-protected CSA have been so far limited to incorporation at the N-terminal position in Fmoc SPPS, to avoid side reactivity with *e.g.* concentrated solutions of piperidine in DMF. While alternative, less nucleophilic bases (*e.g.* 1,8-diazabicyclo(5.4.0)undec-7-ene, DBU) may solve such issues, it remains to be demonstrated.

Another possibly interesting application of these synthetic methods could be to study and quantify the stability of CSA containing peptides in the presence of cellular proteases. Proteolytic profiling against *e.g.* their Asp containing counterparts has the potential to uncover new molecular design criteria for developing engineered proteins and peptidic probes with improved cellular efficacy.

Finally, beyond CSA chemical biology, a better understanding of how Srx interacts with its CSA containing substrates has potential to aid the discovery of small molecule modulators of Srx, and assess its overall potential for drug discovery. While several reports have suggested Srx as a potentially useful target for inhibitor development,^83^ small molecule inhibitors are scarce (*e.g.* J14) and their mode of action remains only partly characterised. One important step towards such inhibitors will be a global assessment of Srx druggability in screening campaigns, for example using fragment screening.

## Author contributions

L. Hayward and M. Baud jointly conceptualised, wrote, and edited this review.

## Conflicts of interest

There are no conflicts of interest to declare.
